# Design and Calibration of a Force/Tactile Sensor for Dexterous Manipulation

**DOI:** 10.3390/s19040966

**Published:** 2019-02-25

**Authors:** Marco Costanzo, Giuseppe De Maria, Ciro Natale, Salvatore Pirozzi

**Affiliations:** Dipartimento di Ingegneria, Università degli Studi della Campania Luigi Vanvitelli, Via Roma, 29, 81031 Aversa, Italy; giuseppe.demaria@unicampania.it (G.D.M.); ciro.natale@unicampania.it (C.N.); salvatore.pirozzi@unicampania.it (S.P.)

**Keywords:** distributed force/tactile sensing, dexterous manipulation, sensor calibration

## Abstract

This paper presents the design and calibration of a new force/tactile sensor for robotic applications. The sensor is suitably designed to provide the robotic grasping device with a sensory system mimicking the human sense of touch, namely, a device sensitive to contact forces, object slip and object geometry. This type of perception information is of paramount importance not only in dexterous manipulation but even in simple grasping tasks, especially when objects are fragile, such that only a minimum amount of grasping force can be applied to hold the object without damaging it. Moreover, sensing only forces and not moments can be very limiting to securely grasp an object when it is grasped far from its center of gravity. Therefore, the perception of torsional moments is a key requirement of the designed sensor. Furthermore, the sensor is also the mechanical interface between the gripper and the manipulated object, therefore its design should consider also the requirements for a correct holding of the object. The most relevant of such requirements is the necessity to hold a torsional moment, therefore a soft distributed contact is necessary. The presence of a soft contact poses a number of challenges in the calibration of the sensor, and that is another contribution of this work. Experimental validation is provided in real grasping tasks with two sensors mounted on an industrial gripper.

## 1. Introduction

The ability of modern service robots to grasp and manipulate objects in a dexterous way is still very far from human manipulation skills. Although complex artificial hands, even anthropomorphic [1,2,3,4,5], have been constructed, the manipulation technology is still at its infancy, not only due to current limitations of both actuation and sensing systems integrated into such complex devices, but also due to the lack of proper control algorithms that should allow the robot to perform manipulation tasks. A detailed review of tactile sensing systems for dextrous robot hands appears in [6]. In particular, only few tactile sensors are able to measure the full 6-D wrench, moreover, sensors that are able to measure also torque (e.g., the 6-axis version of OptoForce) uses an array of 3-D sensors coupled with a rigid surface that limits the frictional torque that the sensor can apply to the grasped object.

One of the main features that any grasping device should possess is the ability to grasp any kind of object as firmly as possible avoiding its slippage even in the presence of external disturbances applied to the object. Such features requires the possibility of modulating the grasping force to allow the robot to manipulate both rigid objects and fragile ones that have to be grasped with the minimum force required to hold them without causing breakage. In multifingered hands, controlling the grasping force requires the proper measurement of contact forces and moments as well as the contact locations at each finger so that external and internal forces can be estimated and properly controlled. These kinds of measurements can be performed with the combined use of tactile and force sensors [7] or by resorting to integrated force/tactile sensors [8,9]. Recently, this kind of sensor has been integrated into commercial parallel grippers for controlling both the linear and rotational slippage of rigid objects of parallelepiped shape [10], demonstrating that it allows the safe grasping of objects under uncertain conditions, namely unknown weight, center of mass and friction, as well as subject to unknown external forces. Underactuated soft robotic hands have been recently used to safely grasp both rigid and fragile objects [11,12]; they exploit the intrinsic compliance of the apparatus to grasp objects with different shapes and stiffnesses.

Grasping is an issue, but how about in-hand manipulation? At a first sight, one might think it can be performed only by multifingered hands. However, currently available anthropomorphic robotic hands still have limited reliability and high complexity, whereas, underactuated soft robotic hands have a limited number of controlled degrees of freedom hence they are mostly used for compliant grasping. That is why manipulation applications mostly adopt simple grippers. Parallel jaw grippers are by far the most widespread, owing to their reliability, low cost and ease of control and integration into standard industrial robots. Such kind of grippers have a limited dexterity, nevertheless researchers have recently demonstrated that they can be used to perform dexterous manipulation actions exploiting the so-called *extrinsic dexterity* concept [13]. In practice, dexterity is provided not only by the degrees of freedom of the grasping device but also by external aids such as gravity or environmental constraints. Examples of in-hand manipulation with these simple devices can be found in [14,15], where a visual feedback was used in an adaptive control algorithm to allow a grasped object rotate in-hand to achieve a given orientation. The same task has been executed by resorting only to force/tactile feedback in [16], where the measurement of both normal and tangential force components together with the torsional moment demonstrated to be effective without the need of any additional external sensors. These experiments, however were carried out only with rigid objects of parallelepiped shape.

In the present paper the complete design and calibration of a new and upgraded version of that force/tactile sensor is presented. The new sensor design starts from the main requirement to manipulate objects of generic shape avoiding both linear and rotational slippage. This goal leads to the need of a soft contact surface so that significant torsional moments can be held by the sensor. To allow application of slipping avoidance algorithms when a soft sensor pad interacts with an object with generic shape, estimation of the contact geometry is essential for the determination of friction model parameters [17], and thus a tactile map with suitable spatial resolution should be designed based on the accuracy requirement on the contact geometry estimation. In this work, it is assumed that objects interacting with the sensor have a curvature radius larger than the one of the sensor pad, therefore estimating contact geometry means an estimation of the normal direction to the contact surface.

Since the tactile map is the sensor output from which all measurements have to be derived, gray-box approaches like in [18] could be adopted. However, physics-based methods were revealed to be effective only in special cases, i.e., interaction with objects with parallelepiped shape, hence a more general calibration procedure has to be sought for. Calibration is of paramount importance in robotics not only for the perception system but also for the robot system itself [19,20]. Furthermore, estimation of the torsional moment poses some constraints on the geometry of the sensor pad. First, the effect of the torsional warping of the deformable layer can be exploited to extract the torsional moment only if its geometry is not axisymmetric. Then, a suitable trade-off between surface curvature and extension of the contact surface must be achieved to ensure proper estimation of force tangential components and torsional moment.

The novel calibration procedure is aimed at enhancing the estimation accuracy especially of the torsional moment component of the contact wrench. It is based on a machine learning approach and, in detail, on the training of a multi-layer feed-forward neural network (FF-NN), and special attention had to be given to the construction of the training set, as the dimension of the input space is 25 while the dimension of the target space is only 6. Therefore, a twofold approach has been followed to ensure proper coverage of both input and target spaces. First, a dedicated graphical user interface (GUI) has been designed to aid the user during the manual calibration of the sensor. The GUI displays in real-time the data acquired by a reference force/torque sensor and those of the force/tactile sensor to be calibrated. Such data are then recorded only if they are considered properly acquired. Specific metrics based on the so-called *limit surface* concept [21] have been purposefully devised to distinguish between admissible and spurious samples. Second, a novel data decimation algorithm in combination with a data compression technique based on the principal component analysis (PCA) has been conceived to ensure the most uniform sampling of both input and target spaces, to avoid unnecessary overfitting of the FF-NN. An important issue in the training of the FF-NN is the large difference in the dimensionality of input and target spaces. In fact, on one hand, accurate estimation of the contact geometry requires high spatial resolution and at least an array of 4×4 taxels. On the other hand, the contact wrench has only six components. Therefore, the data compression pre-processing step is crucial to ensure the minimal complexity of the network architecture. The validation of the calibration procedure is demonstrated by specific validation experiments performed with two sensors mounted on an industrial gripper equipping a robotic arm to grasp different objects. To carry out experiments on the robotic arm, the sensor was interfaced to the control PC via a USB connection in order to avoid the limitations of the gripper communication interface. To reduce the cabling problems, the sensor PCB design is compatible with a wi-fi interface too.

## 2. Design of the Force/Tactile Sensor

This section firstly recalls the requirements and the working principle of a force/tactile sensor for dexterous manipulation applications. Then a discussion about the generalization of the design procedure is presented, which allows the acquisition of an optimal and detailed design of the force/tactile sensor for the integration into commercial grippers.

### 2.1. Requirements for Dexterous Manipulation

The dexterous manipulation is a very challenging robotic task, especially when the objects to manipulate have mechanical properties (e.g., weight, shape, stiffness, friction coefficient) very different from each another and/or not a priori known. In these cases, the use of exteroceptive sensors, able to provide data concerning object properties, is necessary to implement suitable control strategies. The more information that is available, the greater the likeliness of a successful completion of a dexterous manipulation task. The major objective that can be pursued is to design a sensor as similar as possible to human touch, i.e., able to supply different types of information about the manipulated objects at the same time: A tactile map, an estimate of contact forces and moments. Moreover, it should also allow the robot to handle fragile objects without breaking them. To this aim, a first requirement concerns the mechanical interface between the sensing components and the manipulated objects. The use of a rigid interface does not allow manipulation of fragile objects and adaptation to different object shapes. Moreover, a rigid interface allows the measurement of pure torsional moments only by using a flat extended surface and by ensuring that the contact occurs with this flat surface perfectly parallel with the object. As a consequence, a solution with a soft pad as contact interface becomes a mandatory requirement. The design of this soft pad has to provide a domed shape to improve the sensor adaptation to different object shapes. By using a Finite Element Model, in [8] the authors demonstrated how the deformations of the soft cap are related to external force components and that a trade-off between a domed shape with a high and a low curvature radius provides good sensitivity for all force components. The second requirement concerns the transduction method. Whereas the objective is not only the estimation of contact force and moment, the classic solution of a force/torque sensor with mechanical frames with bonded sensing elements (usually strain gages or capacitive sensors) has to be overcome. A distributed measurement of the soft pad deformation due to a contact appears to be the best solution to reconstruct the maximum amount of information about the manipulated object properties. Since this solution is not implementable, the alternative is to measure the deformation of the soft pad in a discrete number of points by using sensing elements spatially distributed on a plane positioned on the bottom side of the pad. These distributed measurements are correlated to the contact state and allow, after a suitable calibration, the reconstruction of several mechanical properties of the object. In particular, in [17] the authors demonstrated how the distributed measurements at the bottom of the soft pad can be used to reconstruct the contact plane pose and consequently the force components in the contact plane, from which physical properties for the contact can then be estimated, such as the friction. Concerning the spatial resolution of the sensing elements, the paper [18] discusses how the estimation of the contact properties depend on this parameter. Moreover, for specific applications (e.g., wires manipulation) the number of sensing elements can also be optimized as shown in [22]. Another requirement is related to both the soft pad shape and the spatial distribution of the sensing elements. In particular, in order to guarantee a sensitivity to external torsional moments applied to the sensor, the soft pad has to be designed with a shape able to transduce the torsional moment into a deformation measurable by the sensing elements. To this aim, the torsional warping phenomenon [23] can be used, by recalling that only the torsion of a non axisymmetric structure allows its generation. As a consequence, the soft pad with a domed top as contact interface should have a non axisymmetric bottom (e.g., a square base). The sensing elements have to be spatially distributed on the area covered by the soft pad base. Additional characteristics to define, common to all sensors, are the working range and the sampling frequency. As deeply discussed in previous papers of the same authors, the force and moment measurement range depends on the hardness of the material used for the realization of the soft pad. Instead, the sampling frequency depends on the number of sensing elements, the interrogation strategy and the communication interface with the main controller [9,24]. For dexterous manipulation tasks the slipping detection and avoidance are fundamental and all techniques presented in literature work much better with higher sampling frequency.

### 2.2. The Working Principle and the Technology

The proposed sensor is based on the working principle firstly presented in [9]. On the basis of requirements described above, the basic idea foresees a suitably designed deformable layer positioned above a discrete number of sensible points (called “taxels”), in order to transduce the external force and moment, applied to the sensor, into deformations, which are measured by the taxels. The taxels, spatially distributed below the deformable layer, provide a set of signals corresponding to a distributed information (called “tactile map”) about the sensor deformations. The whole tactile map allows, after a calibration procedure, to estimate contact force and moment together with information about the orientation of the contact surface and object properties. The taxels have been developed by using optoelectronic technology, and in particular each sensing point is constituted by an emitter and a receiver, mounted side by side, working in reflection mode. The soft pad has been realized by using the silicone molding technology with the molds made with a high resolution 3D printing manufacturing process.

### 2.3. Detailed Design of the Rigid-Flex PCB

**Figure 1:**
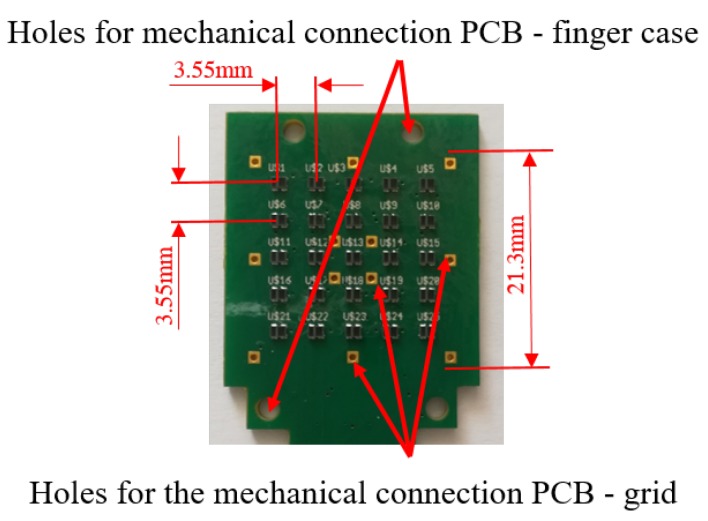
Details of the sensitive area on the Printed Circuit Board (PCB).

**Figure 2:**
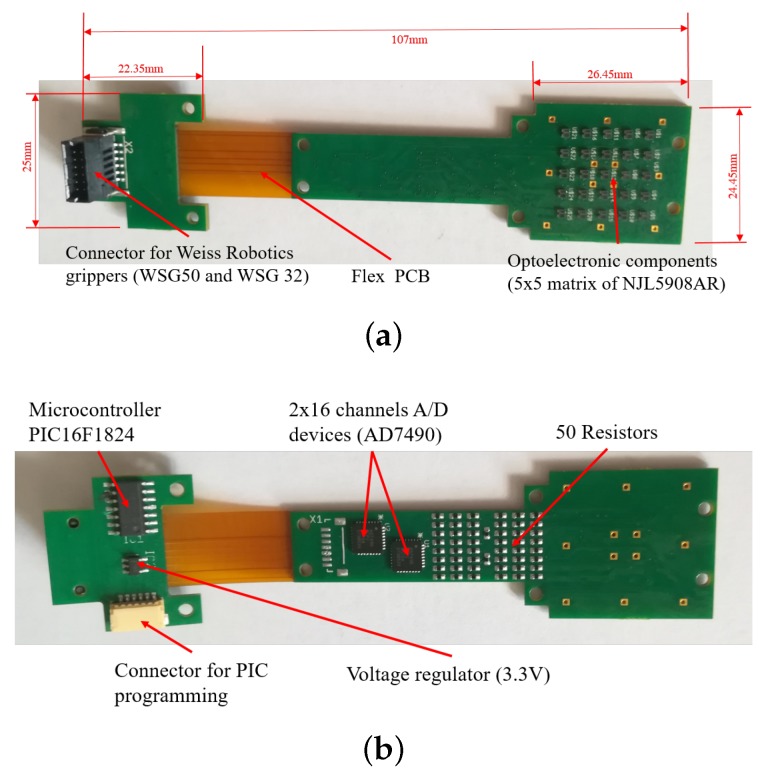
The tactile sensor PCB: a top view with dimensions (**a**) and a bottom view (**b**) with the components highlighted.

Similarly to the initial prototype in [9], the developed sensor is mainly constituted by three components: A Printed Circuit Board (PCB), a rigid grid and a deformable cap. However, for this work, the design of all these components has been optimized on the basis of the requirements discussed in Section 2.1. The first improvement with respect to [9] concerns the type of integrated taxels. In detail, for each taxel, the emitter/receiver couple is here constituted by a unique optoelectronic component: A Surface Mount Technology (SMT) photo-reflector, manufactured by New Japan Radio Co. (San Jose, CA, USA), with part number NJL5908AR. This device integrates in the same package the emitter, an infrared Light Emitting Diode (LED), with a peak wavelength at 920 nm and the receiver, a PhotoTransistor (PT), with a peak wavelength at 880 nm. The surface encumbrance of a single device is 1.06×1.46 mm${}^{2}$. These devices allow the realization of the PCB with a standard robotized pick-and-place procedure, which guarantees the minimization of uncertainties on the taxel positioning and on the relative orientation among the emitter and the receiver of a taxel. These uncertainties negatively affected the sensor performance when separated components were used. Differently from [9], the number of taxels have been increased in order to obtain a sensitive area sufficiently wide to manipulate a larger number of objects. In particular, the optoelectronic section of the PCB integrates 25 taxels, organized in a 5×5 matrix. The device positioning on the PCB has been made in order to obtain a grid of photo-reflectors, with spacings both vertically and horizontally of 3.55 mm among their optical axes. The same distance has been considered on the edges of the optical component matrix, by obtaining a total area to cover with the deformable layer equal to 21.3×21.3 mm${}^{2}$, as shown in Figure 1. The PCB design also foresaw specific holes for the mechanical assembly between the electronic layer and the other sensor components. Figure 1 shows the holes designed to mechanically connect the board to the finger case (via M2 screws) and the holes used to connect the grid and the deformable layer as described in the following. As in previous prototypes, for each taxel, the conditioning electronics is constituted by two resistors: One to drive the LED and a second to transduce the photocurrent measured by the PT into a voltage directly compatible with an Analog-to-Digital (A/D) converter. The same 12-bit A/D converters (manufactured by Analog Devices, with part number AD7490) with 16 channels and a Serial Peripheral Interface (SPI), used in previous works, has been integrated in the PCB design. In this case two converters are needed for the conversion of the 25 taxel signals. A preliminary version of this solution has been presented in [18], where a standard rigid PCB has been realized only with the optoelectronic and the A/D conversion sections. Preliminary tests have been carried out by interrogating the prototype with an external board via the SPI interface. In order to integrate the tactile sensor in a standard parallel gripper, for this work, a microcontroller-based section has been integrated into the PCB design. In particular, an interfacing section constituted by the microcontroller PIC16F1824, manufactured by Microchip Technology, has been added on a separate rigid board, connected to the previously described part via a flexible section. The integration of the microcontroller allows to obtain a fully integrated sensor with a programmable device used to interrogate the sensor via a standard serial interface already available in most commercial grippers. The board is completed by a standard low-noise voltage regulator with an input voltage range up to 12 V (typical range of supply voltage available on commercial grippers) and an output voltage equal to 3.3 V to supply the whole PCB. The use of the rigid-flex technology allows the integration of the PCB into a finger case compatible with the mechanical connection of standard grippers. Figure 2 reports some pictures of the whole rigid-flex PCB, with the dimensions and the description of all components. In the realized prototype, the connector compatible with the sensor port available on the commercial grippers WSG-series, manufactured by Weiss Robotics, has been integrated, in order to provide the 5 V voltage supply to the sensor and for the physical implementation of the serial interface.

### 2.4. Detailed Design of the Deformable Pad

**Figure 3:**
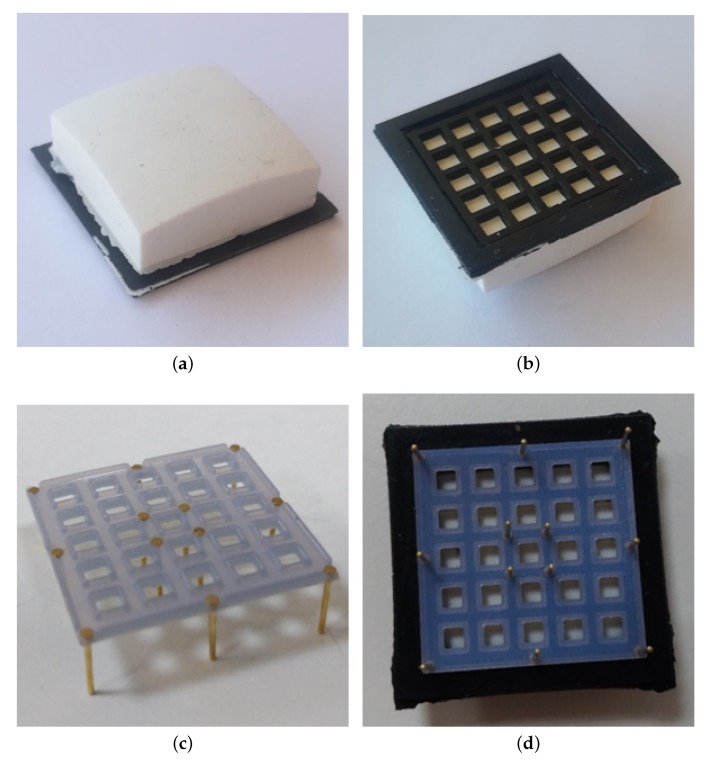
Pictures of deformable layer and rigid grid: Top view (**a**) and bottom view (**b**) of the deformable layer, grid with bonded pins (**c**) and deformable layer assembled with the grid (**d**).

A mechanical structure constituted by the deformable layer and the rigid grid is connected above the PCB. The deformable layer is mainly made of white silicone with a domed top side and a square base, as shown by the picture in Figure 3a. As discussed above, the use of a non axisymmetric shape implies, in presence of torsional moments applied to the deformable layer, the generation of the torsional warping effect, which is measurable by the tactile map and allows the reconstruction of the applied moment. The mechanical properties of the silicone determine the full-scale and the sensitivity of the sensor. The realized prototype uses a shore hardness of 26 A, which corresponds to the working range reported in Section 3. Figure 3b shows the bottom side of the deformable layer, where there are the twenty-five empty cells, which present the ceilings (which in the final assembly are positioned in front of photo-reflectors) made of white silicone, while the walls among the taxels are black, to avoid cross-talk effects. According to the working priciple explained in Section 2.2, when external forces and/or moments are applied to the deformable layer, they produce vertical displacements of the white ceilings for all cells. The distances between the top of photo-reflectors and the white surfaces change, by producing variations of the reflected light and, accordingly, of the voltage signals measured by the PTs.

**Figure 4:**
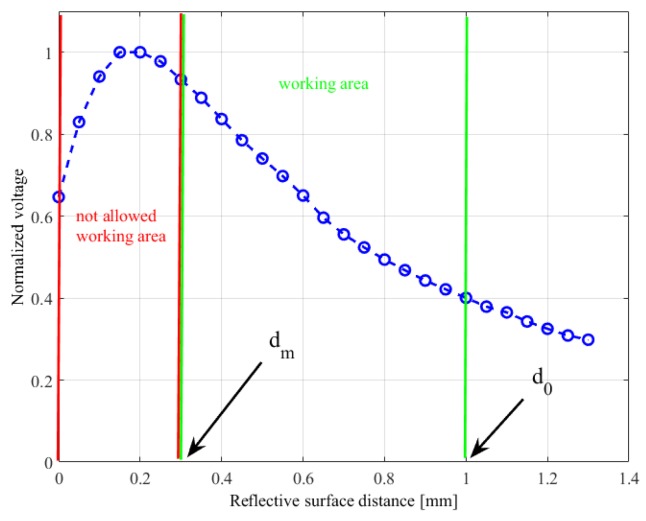
Characteristic for a single taxel: Normalized voltage vs. reflective surface distance.

The addition of the third component (i.e., the rigid grid) became necessary due to the electromechanical characteristic of the optical components. In particular, the NJL5908AR photo-reflector has a non-monotonic characteristic (see Figure 4), which relates the measured voltage to the distance of a reflecting surface positioned in front of the component. As a consequence, the rigid grid has to ensure that the reflecting surface never reaches distances from the component that fall into the non-monotonic area, highlighted by the red bars in Figure 4. Note that this part of the characteristic curve is related to the optical behaviour of the photodetector and it has been obtained without the deformable sensor pad. Taking into account that the height of a component is 0.5 mm, the rigid grid has been designed with a thickness of 0.8 mm. With this choice the minimum reachable distance between a reflecting surface and a photo-reflector is $d_{m}=0.3\;$ mm. On the other side, the silicone layer have been designed so that, in rest condition, the sum of the grid thickness and of the cell walls fixes the white ceilings at an initial distance $d_{0}=1\;$ mm from the emitting surface of the optical components. The integrated design of these two components allows to force the photo-reflectors to work in the monotonic working area, highlighted by the green bars in Figure 4. Considering the high definition needed for the grid realization, a 3D printing manufacturing process based on the PolyJet technology has been selected with a resolution of 16 μm. The grid design foresees holes suitable for housing rigid pins. Figure 3c shows a grid assembled with the pins, which are bonded via a cyanoacrylate-based glue. This assembled grid is then bonded to the deformable layer, by using the same glue, by obtaining the final mechanical cap reported in Figure 3d. The rigid pins that come out of the assembled cap are used to align the cells with the optical components on the PCB, thanks to the mechanical holes available on the board (see Figure 1). After the alignment, the same pins are used to mechanically connect the cap and the PCB, by soldering the rigid pins to the bottom side of the board (see Figure 2b).

**Figure 5:**
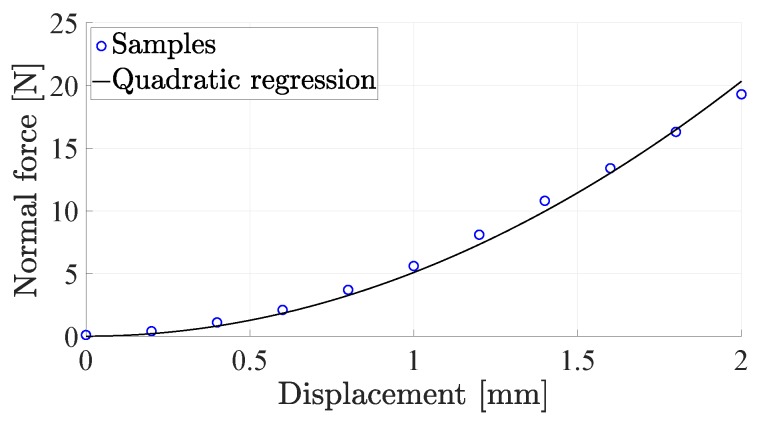
Force–displacement characteristic curve of the sensor pad.

**Figure 6:**
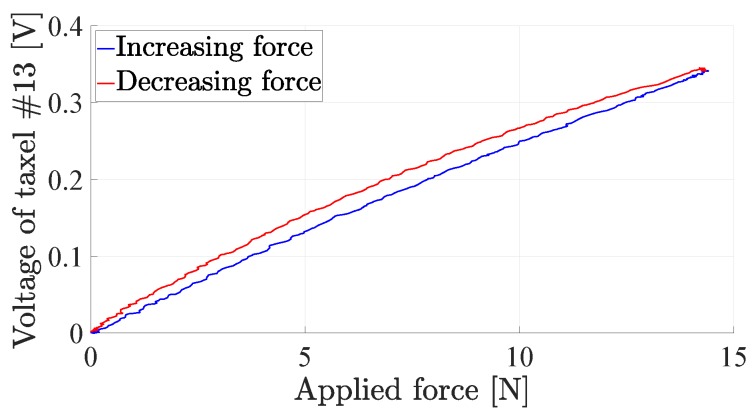
Force–voltage hysteresis curve of a sensor taxel.

The distance-to-voltage characteristic in Figure 4 is not the only source of nonlinearity in the sensor. The hemispherical geometry of the pad yields a nonlinear relationship between the normal force applied to the sensor and the deformation of the sensor pad, due to the variation of the contact area with the force. Figure 5 reports the normal force versus the displacement of the sensor pad tip, when applied with a rigid plane parallel to the sensitive board. The figure shows how a quadratic regression accurately fits the sampled data. Another source of nonlinearity is the material hysteresis that, however, is quite limited (about 5%) due to the specific silicone material selected, as shown in Figure 6, which reports the de-biased voltage of the central taxel versus the applied force.

### 2.5. Integration of the Sensor into a Commercial Gripper

**Figure 7:**
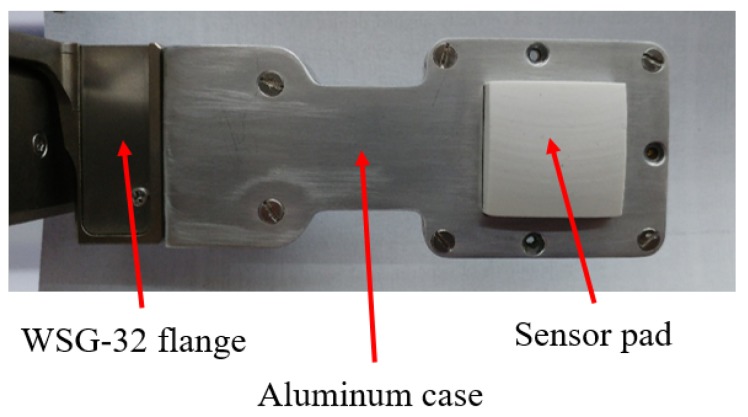
Picture of the sensorized finger fully integrated with the WSG-32 gripper.

**Figure 8:**
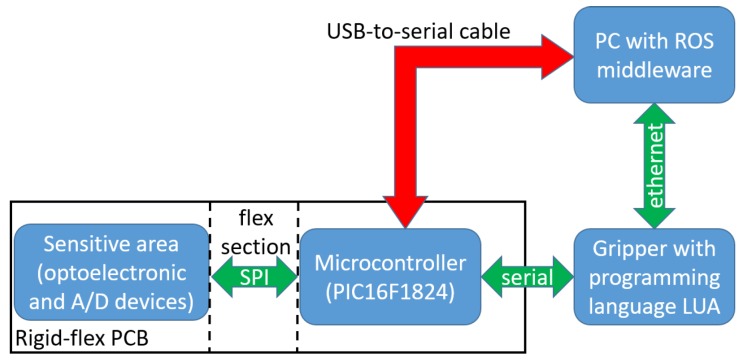
Data flow scheme of possible connections from the sensor to the control PC.

The assembled force/tactile sensor is finally fixed inside an aluminum case suitably designed to house the sensor and for the mechanical connection to the WSG-series flange. Figure 7 reports a picture of the sensorized finger fully integrated with the gripper. The microcontroller section available on the PCB allows two possible connections to exchange data with the main PC. In the fully integrated version, the PCB takes the voltage supply directly from the sensor port available on the WSG-series flange. The same port is used to implement a standard serial communication between the gripper and the sensor. The microcontroller interrogates the A/D converters via an SPI interface and transmits the raw data (2 bytes for each taxel, for a total of 50 bytes) via its serial port. The gripper is programmable by using the LUA programming language, that is an interpreted language suitably designed for embedded applications. This language allows to interrogate the serial port with a maximum baudrate equal to 115,200 bps. The conditioning electronics integrated into the gripper, together with the interpreted language, allows to reach a maximum sampling frequency for all 25 taxels equal to 50 Hz, even though the connection between the gripper and the main PC is implemented by using an Ethernet interface. The second possible connection from the microcontroller to the robot control PC foresees the use of a standard USB-to-serial converter with an external cable, that directly connects the microcontroller to the main PC. In this case, the power supply and the serial transmission are implemented directly from the PC. With this solution the baudrate of the serial port can reach a maximum baudrate equal to 500,000 bps (in this case limited by the microcontroller). This baudrate together with the latency time of the serial port used on the control PC allows to reach a sampling frequency for all 25 taxels equal to 333 Hz. Figure 8 reports a scheme of possible connections currently available. The microcontroller is ready to be interfaced with a serial-to-WIFI adapter, in order to use a wireless connection directly with the PC. This solution will allow the avoidance of limitations related to the serial port latency time with an expected sampling frequency up to 1 kHz. On the control PC, two different ROS nodes have been developed: One to interact with the gripper, if the first solution is selected, and another one to directly interact with the microcontroller in the second case. In both cases the ROS nodes receive raw data (i.e., the 50 bytes acquired by the A/D converters) and the first elaboration consists in the reconstruction of actual voltage values, which are published to be available for the whole ROS network.

## 3. Sensor Calibration

This section illustrates the calibration procedure of the sensor. In [18] the sensor was calibrated with a gray-box model deduced by a FEM analysis. The main limitations of the previous approach are the necessity of an equalizing scaling factor for each voltage and the assumption of a contact plane orientation of θ=0. The equalization is needed to counteract unavoidable different taxel sensitivities due to different gains and operating points of the phototransistor. Moreover, the previous algorithm was able to estimate only the forces and not all the wrench components.

In this paper a new calibration procedure, based on a FF-NN, is proposed. This approach is able to overcame the limitations of the previous procedure, i.e., the parameters are implicitly learned by the FF-NN; the contact plane can be oriented in different positions; the moment vector can be estimated by including it in the target data.

The critical point of the machine learning-based approach is the training data collection. The objective is to estimate the wrench in all possible combinations in a large interval of the contact plane orientation. The dimensionality of the problem is large, so there is a significant risk of missed wrench/orientation combinations in the training set.

Moreover, the dimensionality and the correlation among the inputs, whose number (25 for the sensor in this paper) is significantly larger than the dimension of the target set (6), and the large number of samples acquired during the calibration phase can slow down the training phase and can easily cause unnecessary overfitting.

### 3.1. Construction of the Training Set

**Figure 9:**
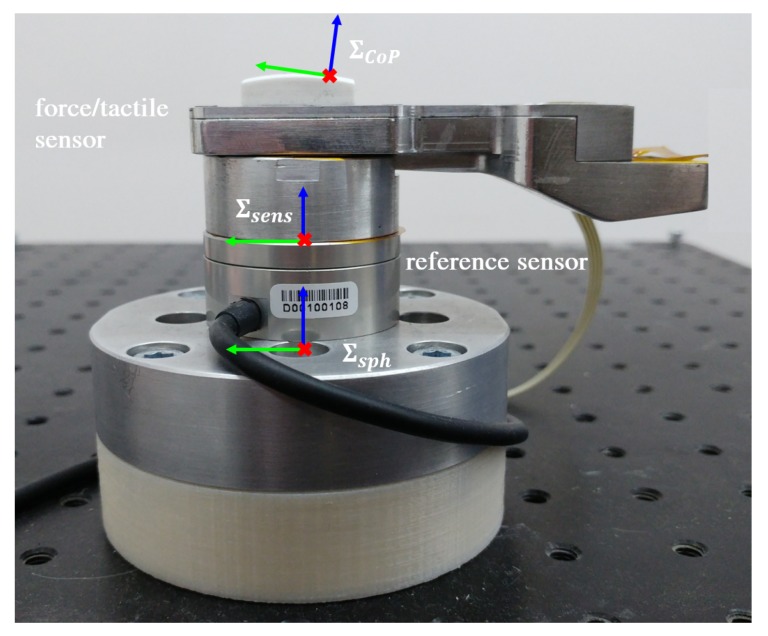
Testbench for sensor calibration.

In order to collect the training set data, the sensor is mounted on a reference force/torque sensor, the Robotous RFT40, as in Figure 9. $\sum_{sens}$ is the reference frame of both sensors; $\sum_{sph}$ is a frame placed in the center of the undeformed silicone sphere; $\sum_{CoP}$ is a frame placed in the center of pressure (CoP) of the contact area with the *z* axis normal to the contact plane.

Data generation is made by an operator who applies forces and moments by touching the sensor with an object. The target wrench and the input tactile voltages are recorded synchronized through the ROS network (see Section 2.5).

In order to ensure a good training set, the input space (and consequently the target space) has to be properly covered. Furthermore, bad data should be avoided, e.g., samples during slipping of the object on the sensor pad surface or during the relaxing phase of the deformable layer.

**Figure 10:**
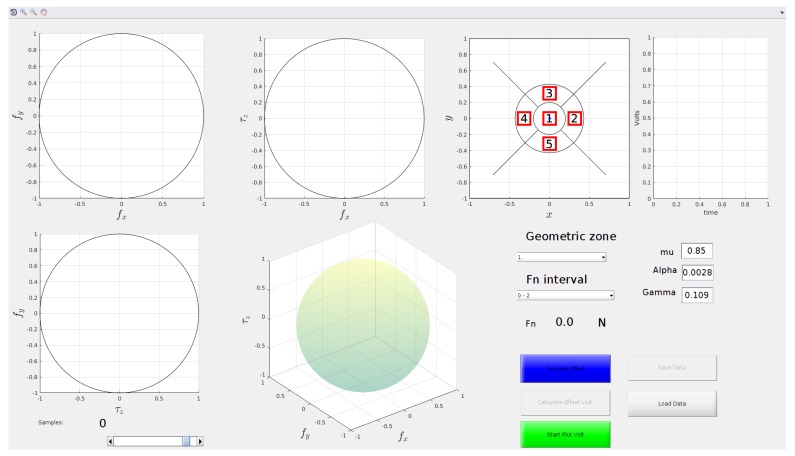
Graphical user interface (GUI) used in the calibration procedure.

These issues are tackled by resorting to a dedicated Matlab GUI (Figure 10). The user interface displays in real-time the calibration data acquired. The visualization of the samples is carried out using the limit surface (LS) theory [21], which is an extension of the Coulomb friction model to the case of roto-translational slippage. The LS gives information about the maximum force and torsional moment that can be applied before a slippage occurs, it is a surface defined in the 3D space of the two tangential force components and the torsional moment (the component of the contact moment along the direction normal to the contact surface). When the wrench is inside this surface no slippage occurs, otherwise, there is relative motion between the two contacting surfaces. The maximum pure tangential force (that is the component of the force tangential to the contact surface) and torsional moment are given by:

(1) $$\begin{array}{cc} {f_{t}}_{\max} & =\mu f_{n}; \end{array}$$

(2) $$\begin{array}{cc} {\tau_{n}}_{\max} & =\alpha f_{n}^{\gamma +1}; \end{array}$$

where $f_{n}$ is the component of force normal to the contact frame, *μ* is the classical Coulomb friction coefficient, *α* and *γ* are parameters of the maximum torque model [25]. All parameters are experimentally estimated through the procedure described in [16].

The GUI visualizes the 3D space of tangential force and torsional moment normalized with respect to $f_{t_{\max}}$ and $\tau_{n_{\max}}$, respectively, using four different plots: A 3D plot and three separate plots one for each view from each coordinate axis. In this normalized space, the LS is approximated as a unit sphere centered in the origin drawn in the 3D plot. This method is useful to discard samples acquired in any slipping phase, namely, samples outside the LS are not included into the training set.

This decision about data inclusion cannot be directly taken based on the measured wrench referred to the sensor frame $\sum_{sens}$. In fact, the LS is defined based on the wrench referred to the $\sum_{CoP}$ frame. The homogeneous transformation matrix expressing the pose of the CoP frame with respect to the sensor frame is estimated considering the tactile map. First of all, the centroid of the tactile map is calculated as

(3) $$\begin{array}{cc} x_{C} & =\frac{\sum_{i=1}^{25}x_{i}\Delta v_{i}}{\sum_{i=1}^{25}\Delta v_{i}}; \end{array}$$

(4) $$\begin{array}{cc} y_{C} & =\frac{\sum_{i=1}^{25}y_{i}\Delta v_{i}}{\sum_{i=1}^{25}\Delta v_{i}}; \end{array}$$

where ($x_{i}$,$y_{i}$) are the coordinates of the *i*th taxel and $\Delta v_{i}$ is the difference between the actual voltage value and the voltage value in rest conditions. The centroid is also plotted in the GUI in a separate plot to help the user understand where he/she currently is touching the sensor. The CoP is considered located in the point on the contact surface corresponding to neglecting the deformation of the sphere (consider that this computation is simply aimed at helping the operator in the calibration procedure). Hence, the coordinates of the CoP with respect to the $\sum_{sph}$ frame are

(5) $$p_{CoP}^{sph}=\left[\begin{array}{c} x_{C}\\ y_{C}\\ \sqrt{R^{2}-x_{C}^{2}-y_{C}} \end{array}\right]$$

where R=50 mm is the sphere radius. Given the distance between $\sum_{sph}$ and $\sum_{sens}$ (20 mm) it is trivial to find the coordinates of the CoP with respect to the sensor frame ($p_{CoP}^{sens}$).

The orientation of the contact plane is basically given by the normal vector to the contact plane. Since the GUI is just an aid for the operator, the contact plane can be well approximated as tangent to the sphere. So the normal unit vector can be calculated with respect to the sphere frame as

(6) $${\hat{n}}_{CoP}^{sph}=\frac{1}{R}p_{CoP}^{sph}.$$

Choosing the sphere frame aligned to the sensor frame, this normal vector has the same components in the sensor frame and it is selected as the *z*-axis of the contact frame. The *x* and *y* axes of the contact frame can be trivially choosen as the projection of the same axes of sensor frame on the contact plane (conveniently normalized). The computed axes can be organized into a rotation matrix $R_{CoP}^{sens}$ and, finally, the homogenouse transformation matrix of the contact frame is

(7) $$T_{CoP}^{sens}=\left[\begin{array}{cc} R_{CoP}^{sens} & p_{CoP}^{sens}\\ 0^{T} & 1 \end{array}\right].$$

Finally, by inverting the last matrix, it is possible to find the force and moment vectors in the contact frame

(8) $$\begin{array}{cc} f^{CoP} & =R_{sens}^{CoP}\;f^{sens}, \end{array}$$

(9) $$\begin{array}{cc} \tau^{CoP} & =R_{sens}^{CoP}\;\tau^{sens}+p_{sens}^{CoP}\times f^{CoP}. \end{array}$$

With the LS aid, the operator can visualize only the tangential forces and the torsional moment. It is not possible to see variations in the normal force and in the contact plane orientation. Moreover, it is impossible to include such information in the plot because it is already a 3D plot and plots with higher dimensions are impossible to easily visualize. To overcome this problem, in the GUI the operator can select a target interval for the normal force among a set of predefined intervals. In addition, the GUI shows the base surface of sensor divided in polar areas (see Figure 10). Given the centroid position, a polar area is uniquely defined. In the same manner, given the normal force value, an interval of forces is defined. In this way it is possible to define various 3D spaces, one for each possible combination of the normal force interval and polar area. The task of the operator is to cover all these 3D spaces with samples, and the program will automatically discard bad samples.

### 3.2. Training Set Pre-Processing

The pre-processing step has a twofold aim. It first reduces the dimension of the input space, that is significantly larger (25) than the target space dimension (6), through a data compression technique, e.g., the Principal Component Analysis (PCA). Furthermore, it tries to make the sample density of the training set as uniform as possible via a new decimation algorithm.

The motivation for such an algorithm is that samples are often collected so that there are zones of the training set with a very high density compared to others. This is typical when forces are low, e.g., the operator is at the beginning of a maneuver. So there are a lot of samples that add few new information to the dataset. This can cause a useless increase in the computational load and can encourage the learning algorithm to specialize the model towards the behaviour in these high density zones. Therefore, these samples should be removed.

The number of samples is reduced through a novel bubble-based decimation algorithm described hereafter in a general case. The idea is to fix a maximum density for the samples in the input space. Let *N* be the total number of samples and the couple $\left(v_{i},w_{i}\right)$ the *i*th sample with input $v_{i}\in R^{m}$ and target $w_{i}\in R^{t}$, the training set is:

(10) $$T_{s}=\left\{\left(v_{i},w_{i}\right),\;i\in I_{T_{s}}\right\}.$$

where

(11) $$I_{T_{s}}=\left\{1,\ldots ,N\right\}.$$

Note that in the particular case of study $v_{i}\in R^{25}$ and $w_{i}\in R^{6}$. The main idea is to define a bubble in the space of the inputs such that, centering the bubble in a sample, no other sample is in the bubble. In other words, the objective is to find a subset $T_{s}^{*}$ of $T_{s}$ such that

(12) $$T_{s}^{*}=\left\{\left(v_{i},w_{i}\right)\in T_{s},i\in I_{T_{s}^{*}}\right\},$$

where

(13) $$I_{T_{s}^{*}}=\{j\in I_{T_{s}}:∥v_{j}-v_{k}∥>r\;\forall k\in I_{T_{s}},\;k\neq j\}$$

being *r* the radius of the bubble. In this way the maximum density in the input space will be of one sample per bubble.

A Matlab function that implements the decimation algorithm is the following:

**function** [ inputs , targets ,mask_keep ] . . .
    = decimation( inputs , targets , radius )
*% Bubble-based decimation function ,*
*% Ts = {( inputs _ i , targets _ i ) }*
     
*%Initialization*
radius_square = radius ^2;
mask_keep = false ( 1 , **size** ( inputs , 2 ) ) ;
mask_not_computed = ~mask_keep ;
*%Repeat  until  process  all  samples*
**while any** (mask_not_computed )
  actual_index = **find** (mask_not_computed , 1 ) ;
  mask_keep ( actual_index ) = true ;
  mask_not_computed ( actual_index ) = false ;
  mask_not_computed (mask_not_computed ) = . . .
   **sum**( ( inputs ( : ,  mask_not_computed ) . . .
      − inputs ( : , actual_index ) ) .^2 . . .
     ) > radius_square ;
**end**
*%select  only  good  samples*
inputs = inputs ( : , mask_keep ) ;
targets = targets ( : , mask_keep ) ;
**end**


The bubble-based decimation can be applied to an heterogeneous input space too, e.g., made of inputs of a different scale. In that case it is necessary to have a pre-normalization of the input data.

Considering that for each normal force interval and polar area the voltage map has to be rather different, this algorithm can be applied separately on the data of each 3D space defined in Section 3.1. In this way the computational load of the decimation is reduced.

**Figure 11:**
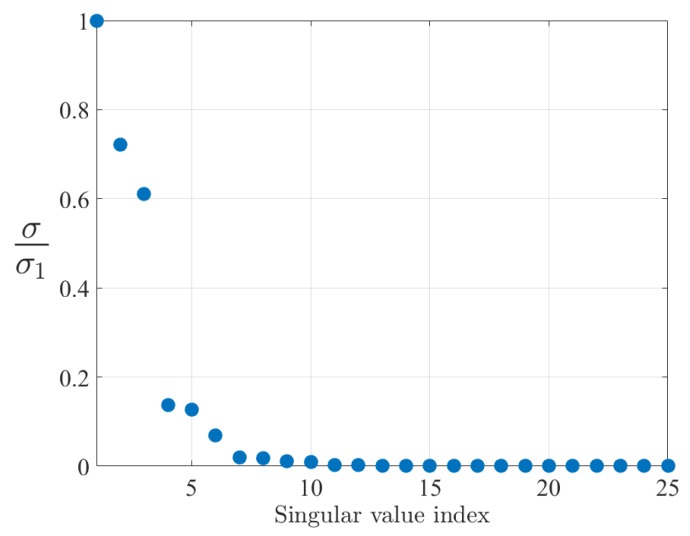
Normalized singular values of the Principal Component Analysis (PCA) of the training set.

The second goal of the preprocessing step is the reduction of the input space dimension and this is achieved by applying a Principal Component Analysis (PCA) technique. Figure 11 reports the plot of the singular values of the input covariance. The plot is normalized with respect to the maximum singular value and, after 15 components, the singular values are below the 0.1%. The choice made here is to take into account the first r=15 components.

Let $U\in R^{m\times r}$ be the matrix of the first *r* singular vectors, the *i*th compressed input will be

(14) $$v_{i}^{*}=U^{T}v_{i}.$$

Eventually, the reduced and compressed training set is used to train a FF-NN with *h* hidden layers, each made of $n_{l}$ neurons, with l=1…h.

### 3.3. FF-NN Training

**Figure 12:**
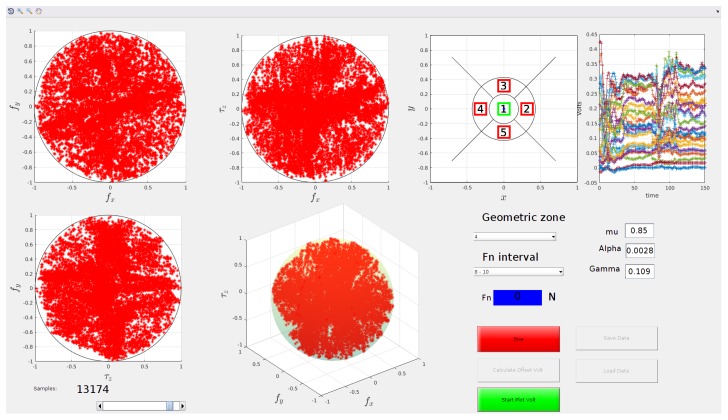
Training set visualized on the GUI after the bubble-based decimation.

After collecting the training set according to the procedure described in Section 3.1, the bubble-based decimation algorithm of Section 3.2 has removed the 53.45% of samples. The GUI can be used to visualize the reduced set in order to graphically view the quality of the decimation and the degree of coverage of the target space. If the decimation had removed too many samples there would be holes in one or more of the plots in the GUI. Figure 12 shows the $T_{s}^{*}$ data in the GUI, the samples are visualized for a normal force in the interval [8,10] and for a centroid position inside zone 4. It is possible to see that, despite the data decimation, the 3D space is still well covered.

**Figure 13:**
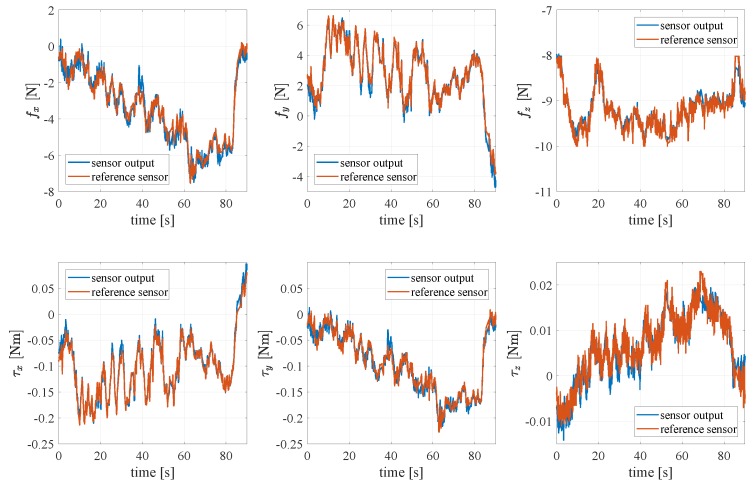
Result of the feed-forward neural network (FF-NN) fitting on the whole acquired training set before decimation.

The data are finally used to train a FF-NN. The network is made of six hidden layers in each one with 30 neurons and a sigmoidal activation function, contrariwise the output layer has a linear activation function and six neurons. Figure 13 shows the FF-NN fitting on the training data in a certain range. The quality of the reconstruction of all wrench components is satisfactory.

## 4. Experimental Validation

This section presents the experimental validation of the proposed sensor and algorithms for its calibration in different experiments with two sensors mounted on the WSG-50 parallel gripper grasping an object in several configurations so as to assess the quality of calibration of all components of the wrench.

**Figure 14:**
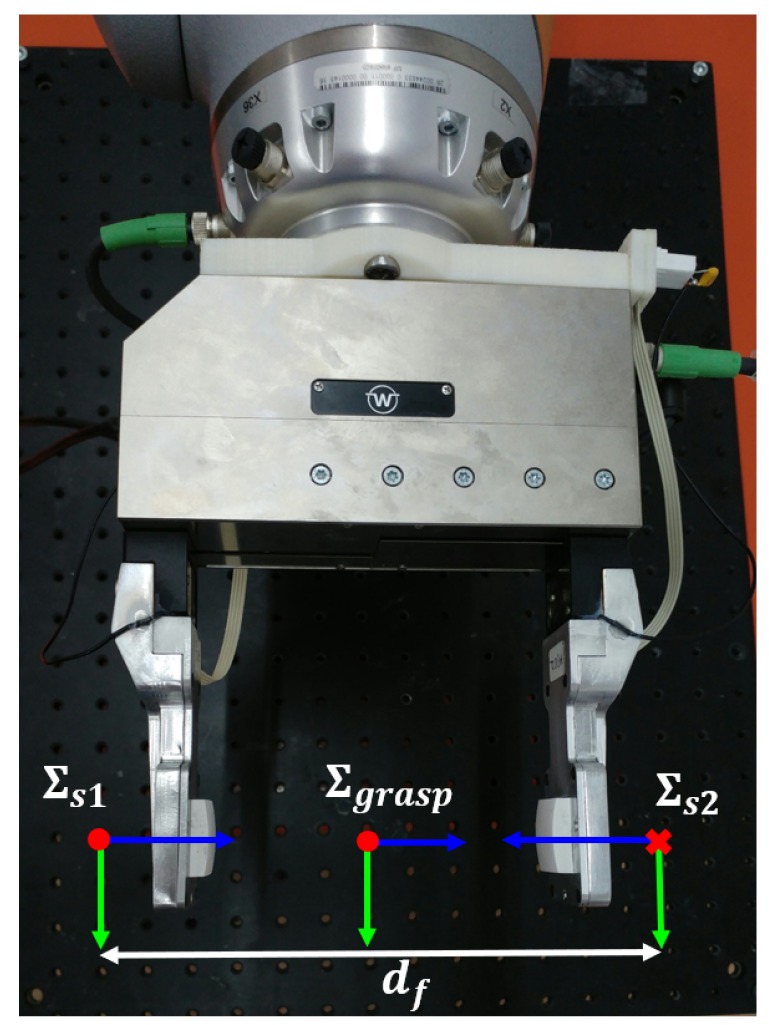
Definition of the grasp frame $\sum_{grasp}$.

The sensors are mounted on the parallel gripper as shown in Figure 14. The external forces applied affect both sensors in a non-symmetric manner (depending on the grasped object orientation and shape). In order to measure the external forces on the object it is necessary to combine the measures of both sensors. Let $\sum_{s1}$ and $\sum_{s2}$ be the frame of each sensor, and $\sum_{grasp}$ be the grasp frame that is located at the center of the fingers as shown in Figure 14. The relative position of these frames is not constant, but it depends on the position of the gripper fingers.

Choosing the grasp frame aligned with the sensor 1 frame, the relative position of the frames is given by

(15) $$\begin{array}{cc} R_{s1}^{grasp} & =I_{3}, \end{array}$$

(16) $$\begin{array}{cc} R_{s2}^{grasp} & =\left[\begin{array}{ccc} -1 & 0 & 0\\ 0 & 1 & 0\\ 0 & 0 & -1 \end{array}\right], \end{array}$$

(17) $$\begin{array}{cc} p_{s1}^{grasp} & =\left[\begin{array}{c} 0\\ 0\\ -d_{f}/2 \end{array}\right], \end{array}$$

(18) $$\begin{array}{cc} p_{s2}^{grasp} & =\left[\begin{array}{c} 0\\ 0\\ d_{f}/2 \end{array}\right], \end{array}$$

where $d_{f}$ is the distance between fingers which depends on gripper state. Let $s_{i}$ be the *i*th sensor, the wrench of each finger can be expressed in the grasp frame as

(19) $$\begin{array}{cc} f_{s_{i}}^{grasp} & =R_{s_{i}}^{grasp}f_{s_{i}}^{s_{i}} \end{array}$$

(20) $$\begin{array}{cc} \tau_{s_{i}}^{grasp} & =R_{s_{i}}^{grasp}\tau_{s_{i}}^{s_{i}}+p_{s_{i}}^{grasp}\times f_{s_{i}}^{grasp} \end{array}$$

Finally, the external wrench in the grasp frame is simply the sum of the components of each finger in the grasp frame, in fact, the grasp matrix is simply constituted by two identity matrices owing to the so-called virtual stick concept [26], i.e.,

(21) $$\left[\begin{array}{c} f^{grasp}\\ \tau^{grasp} \end{array}\right]=\left[\begin{array}{cc} I_{6} & I_{6} \end{array}\right]\left[\begin{array}{c} f_{s_{1}}^{grasp}\\ \tau_{s_{1}}^{grasp}\\ f_{s_{2}}^{grasp}\\ \tau_{s_{2}}^{grasp} \end{array}\right].$$

Note that since the *z*-axes of the three frames are aligned to the same straight line, the variable $d_{f}$ affects only the two moments $\tau_{x}$ and $\tau_{y}$ and not the others components of the wrench.

### 4.1. Reconstruction of the Normal Force Component

**Figure 15:**
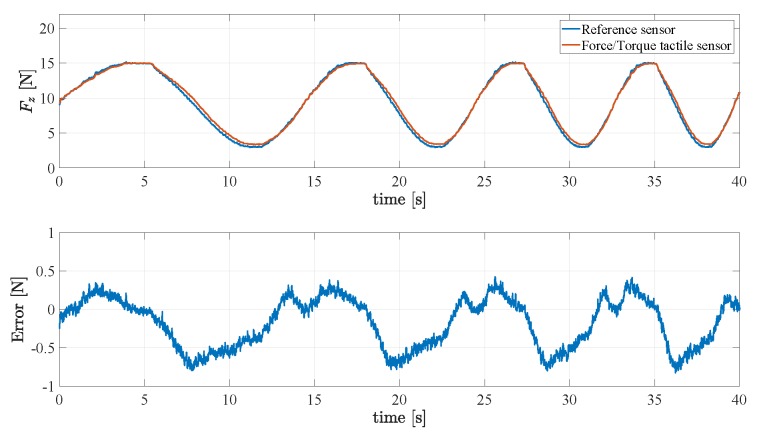
First experiment: Assessment of the reconstruction of the normal force component.

A first validation experiment is aimed at assessing the quality of the reconstruction of the normal force component. The reference force sensor is grasped by the gripper applying a chirp force signal from 0.05 Hz to 0.1 Hz in 40 s to the fingers. The estimated normal force of a finger is then compared to the normal force measured by the reference sensor. The signals and the corresponding error are reported in Figure 15, which shows a maximum error of about 0.7 N for a maximum force of 16 N.

### 4.2. Reconstruction of the Tangential Force Components

**Figure 16:**
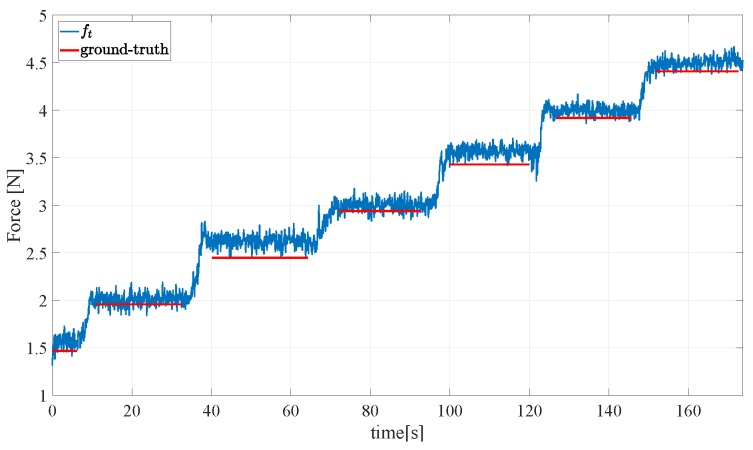
Second experiment: Assessment of the reconstruction of the grasped object weight—first grasp.

**Figure 17:**
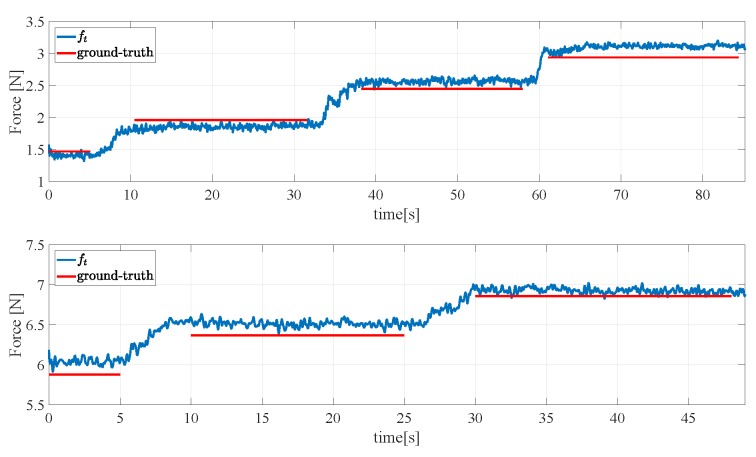
Second experiment: Assessment of the reconstruction of the grasped object weight—second grasp.

**Figure 18:**
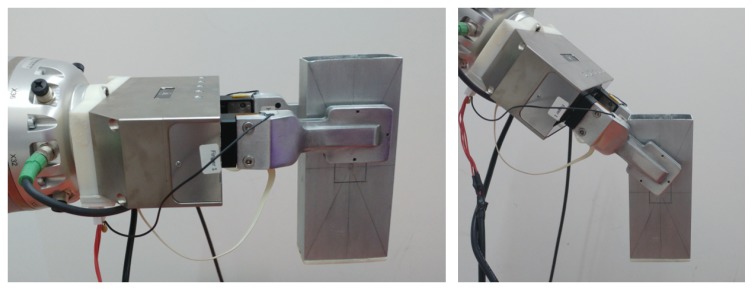
Second experiment: First grasp (**left**) and second grasp (**right**) for the assessment of the tangential force components accuracy.

Figure 16 and Figure 17 show the result of a second experiment, where an empty aluminium box is grasped with two orientations of the gripper, one holding the object as in Figure 18 (left) and the second one holding the object as in Figure 18 (right) so as to generate tangential components along both $x_{s}$ and $y_{s}$. Then, the box is filled in with weights of 0.49 N released one after the other. Figure 16 reports the ground truth weights (red bars) compared to estimated weights in terms of the total tangential force in the grasp frame (indicated with $f_{t}$ in the figure legends). Taking into account that the weight of the empty box is 1.5 N, the accuracy of the sensor is quite satisfactory as the largest error is about 0.2 N and each measured step corresponds to about 0.5 N. The same experiment has been repeated by rotating of the gripper 45${}^{\circ }$ and the results in Figure 17 (top) show a similar behaviour and accuracy. Figure 17 (bottom) refers to a similar trial by starting the filling process of the box previously filled in for a total initial weight of 5.9 N, so as to reach higher tangential forces. The errors keep below 0.2 N.

### 4.3. Assessment of Sensor Sensitivity and Dynamic Range

**Figure 19:**
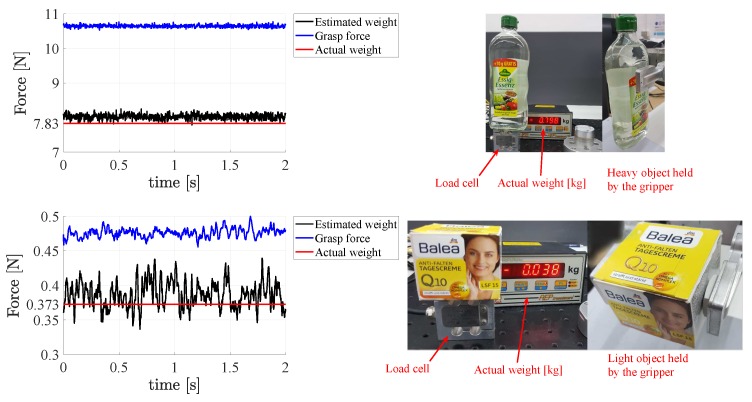
Third experiment: Assessment of sensor sensitivity (**bottom**) and dynamic range (**top**).

A third experiment has been carried out to assess the sensor sensitivity and to demonstrate that it is able to measure very low forces and thus it can be exploited to grasp both light objects with low gripping forces and heavy objects that require large forces to be held. A heavy glass bottle is grasped by the gripper and the weight estimated by the sensors is compared to the actual weight in Figure 19 (top) to show how the sensor behaves close to its full scale range. To demonstrate that the sensor can actually measure low forces with an accuracy high enough to effectively grasp an object with correspondingly light gripping force, a light and empty cardboard box is grasped and its estimated weight is compared to the actual one. Figure 19 (bottom) shows the result of the test, which confirm that the light grasping force of about 0.5 N does not cause any noticeable deformation of the box. It should be highlighted that the estimated weight of about 0.4 N is computed as in (21) and this means that each sensor is able to measure half of such value.

### 4.4. Reconstruction of the Contact Plane Orientation

**Figure 20:**
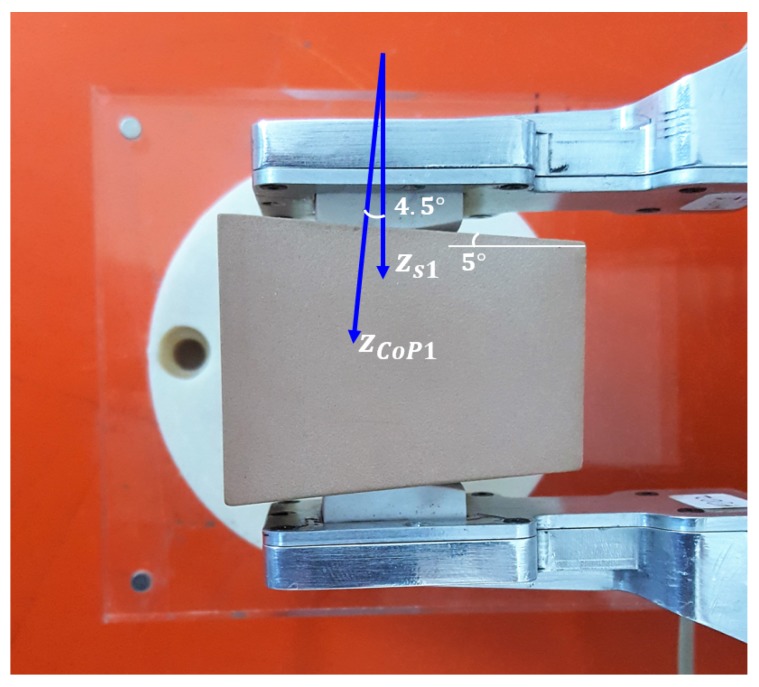
Fourth experiment: Grasp to validate the contact geometry estimation capability.

**Figure 21:**
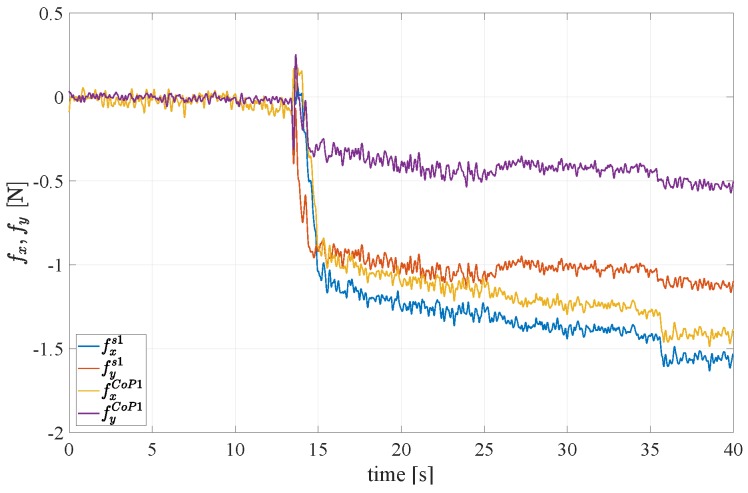
Fourth experiment: Force components rotated into the CoP frame.

**Figure 22:**
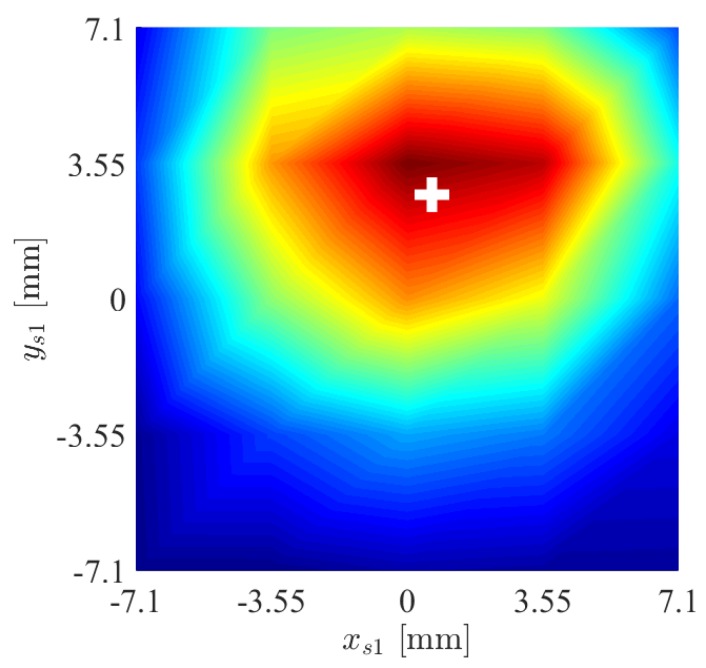
Fourth experiment: Tactile map and corresponding centroid (white cross).

A fourth experiment is devoted to evaluate the capability of the force/tactile sensor to estimate the contact force components in the CoP frame as defined in Section 3. An object with non parallel faces is grasped between the fingers as shown in Figure 20. It is clear that in the CoP frame, the tangential components should be lower than those in the sensor frame, on the contrary the normal component in the sensor frame is lower than that in the CoP frame. This expectation is confirmed by the results reported in Figure 21. To quantify the accuracy in the estimation of the contact plane orientation, the contact normal is estimated according to Equation (6) based on the centroid of the tactile map, shown in Figure 22 by spatially interpolating the taxel values. Note that the small *x* component of the centroid can be attributed to a slight misalignment of the gripper with respect to the horizontal table where the object is placed. The estimated angle between the normal direction and the $z_{s1}$ direction is equal to 4.5${}^{\circ }$, compared to an actual value of 5${}^{\circ }$. It is evident that computation of the ratio between normal and tangential contact force components is significantly affected by the angle between the contact normal and the *z* axis of the sensor frame. Such ratio is at the basis of any strategy for slipping avoidance based on friction models.

### 4.5. Reconstruction of the Torsional Moment

**Figure 23:**
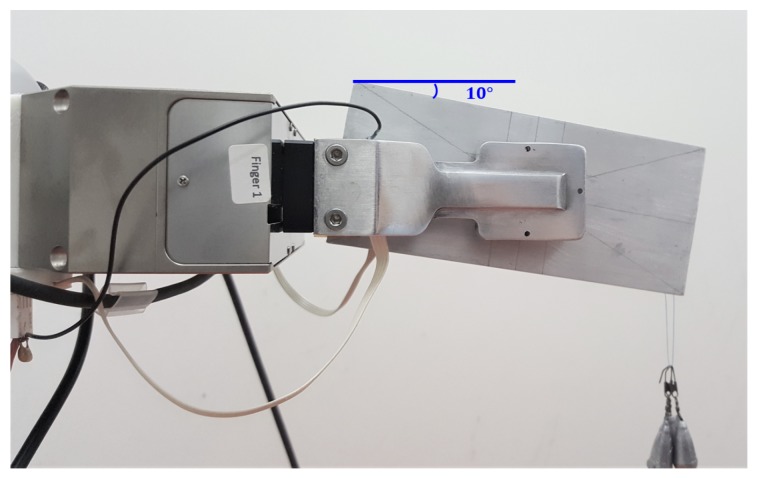
Fifth experiment: Grasp for the validation of the calibration of the torsional moment component.

**Figure 24:**
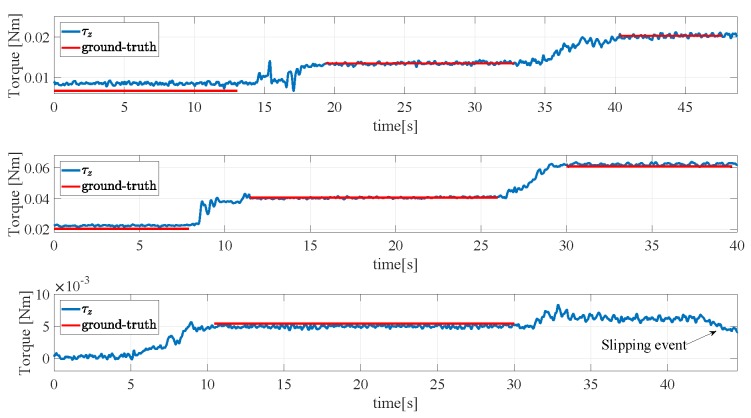
Fifth experiment: Validation of the calibration of the torsional moment component, with $f_{n}=5\;$ N (**top**), $f_{n}=15\;$ N (**middle**), $f_{n}=2.5\;$ N (**bottom**).

**Figure 25:**
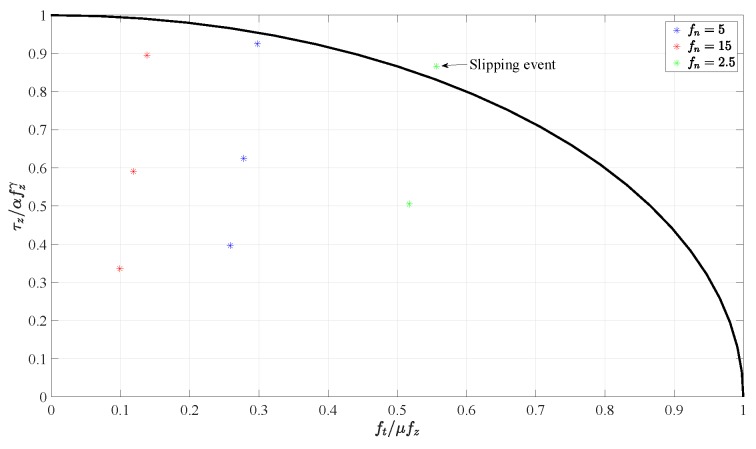
Fifth experiment: Tangential force and torsional moment couples with respect to the limit surface.

A fifth experiment is devoted to validate the calibration algorithm for the reconstruction of the torsional moment component. An aluminium box with an initial weight of 1.22 N has been grasped as shown in Figure 23 in its center of gravity. Then, different weights have been hung at an extremity to apply a torsional moment to the sensors. The results are reported in Figure 24. The top subplot refers to a normal grasp force of 5 N and three weights each of 0.098 N have been hung one after the other corresponding to steps in the torsional moment of about 0.0068 Nm. This final condition corresponds to the image reported in Figure 23 where the 10${}^{\circ }$ rotation of the aluminium box is caused by the torsional deformation of the sensor pad, and not by a slippage. In the trial shown in the middle subplot, the grasp force is increased to 15 N to allow holding larger torsional moment. Three weights of 0.29 N have then been hung to the box extremity reaching a total torsional moment of about 0.061 Nm with steps of 0.02 Nm. In the last trial reported in the bottom subplot the grasp force is reduced down to 2.5 N and after hanging a second weight of 0.098 N a slipping event occurs as demonstrated by the sudden drop of the measured torsional moment. The tangential forces and torsional moments measured in the whole fourth experiment are normalized with respect to (1) and (2), respectively, and reported together with the limit surface in Figure 25. The red and blue dots are all inside the limit surface and in fact no slipping occurs, while the green dot outside the limit surface corresponds to the slipping event indicated in the bottom subplot of Figure 24.

## 5. Conclusions

This paper has presented the detailed design and experimental characterization of a force/tactile sensor able to measure distributed contacts and estimate contact force and torsional moments to be used for robotic dexterous manipulation tasks. The mechanical interface of the device is a soft pad of silicone so as to adapt to different object shapes and hold high torsional moments. The sensitive part, based on optoelectronic technology, is exploited not only to estimate the total contact wrench but also to detect the orientation of the contact surface essential to correctly detect friction force, a relevant quantity in any dexterous manipulation control algorithm. The complete design of both the transducer and the interface electronics for integration into industrial grippers is discussed together with a novel calibration procedure aided by a specifically designed GUI that is aimed at ensuring a proper coverage of the training set necessary for the neural-network training selected as the calibration algorithm. The effectiveness of the calibration is experimentally validated through a number of trials carried out on a standard parallel gripper equipped with two sensorized fingers mounted on a robotic arm.

Future developments will be devoted to exploit the tactile map correlated to the force measurements for texture recognition through machine learning algorithms. This ability is of particular interest in service robotics applications where the robot has to recognize the object it is interacting with. Furthermore, an integrated force/tactile sensor is the enabling technology for executing not only grasping of unknown objects with the minimum required force to avoid slippage, but also for carrying out more complex in-hand manipulation actions, such as controlled sliding maneuvers. The possibility to control the orientation of the manipulated object by simply acting on the gripping force allows the robot to adopt only simple parallel grippers to perform such dexterous operations. This can pave the way to the adoption of robots in many application fields which require sophisticated manipulation abilities, such as logistics and household environments.
